# Assessment of Remote Myocardium Heterogeneity in Patients with Ventricular Tachycardia Using Texture Analysis of Late Iodine Enhancement (LIE) Cardiac Computed Tomography (cCT) Images

**DOI:** 10.1007/s11307-018-1175-1

**Published:** 2018-03-13

**Authors:** Antonio Esposito, Anna Palmisano, Sofia Antunes, Caterina Colantoni, Paola Maria Vittoria Rancoita, Davide Vignale, Francesca Baratto, Paolo Della Bella, Alessandro Del Maschio, Francesco De Cobelli

**Affiliations:** 1 Clinical and Experimental Radiology Unit, Experimental Imaging Center, San Raffaele Scientific Institute, Via Olgettina 60, 20132 Milan, Italy; 2 Vita-Salute San Raffaele University, Milan, Italy; 3 Images Post-Processing and Analysis Unit, Experimental Imaging Center, San Raffaele Scientific Institute, Milan, Italy; 4 University Centre for Statistics in the Biomedical Sciences (CUSSB), Vita-Salute San Raffaele University, Milan, Italy; 5 Arrhythmia Unit and Electrophysiology Laboratories, San Raffaele Scientific Institute, Milan, Italy

**Keywords:** Cardiac computed tomography, Extracellular volume fraction, Late iodine enhancement, Myocardial characterization, Heterogeneity

## Abstract

**Purpose:**

Diffuse remodeling of myocardial extra-cellular matrix is largely responsible for left ventricle (LV) dysfunction and arrhythmias. Our hypothesis is that the texture analysis of late iodine enhancement (LIE) cardiac computed tomography (cCT) images may improve characterization of the diffuse extra-cellular matrix changes. Our aim was to extract volumetric extracellular volume (ECV) and LIE texture features of non-scarred (remote) myocardium from cCT of patients with recurrent ventricular tachycardia (rVT), and to compare these radiomic features with LV-function, LV-remodeling, and underlying cardiac disease.

**Procedures:**

Forty-eight patients suffering from rVT were prospectively enrolled: 5/48 with idiopathic VT (IVT), 23/48 with post-ischemic dilated cardiomyopathy (ICM), 9/48 with idiopathic dilated cardiomyopathy (IDCM), and 11/48 with scars from a previous healed myocarditis (MYO). All patients underwent echocardiography to assess LV systolic and diastolic function and cCT with pre-contrast, angiographic, and LIE scan to obtain end-diastolic volume (EDV), ECV, and first-order texture parameters of Hounsfield Unit (HU) of remote myocardium in LIE [energy, entropy, HU-mean, HU-median, standard deviation (SD), and mean absolute deviation (MAD)].

**Results:**

Energy, HU mean, and HU median by cCT texture analysis correlated with ECV (rho = 0.5650, rho = 0.5741, rho = 0.5068; *p* < 0.0005). cCT-derived ECV, HU-mean, HU-median, SD, and MAD correlated directly to EDV by cCT and inversely to ejection fraction by echocardiography (*p* < 0.05). SD and MAD correlated with diastolic function by echocardiography (rho = 0.3837, *p* = 0.0071; rho = 0.3330, *p* = 0.0208). MYO and IVT patients were characterized by significantly lower values of SD and MAD when compared with ICM and IDCM patients, independently of LV-volume systolic and diastolic function.

**Conclusions:**

Texture analysis of LIE may expand cCT capability of myocardial characterization. Myocardial heterogeneity (SD and MAD) was associated with LV dilatation, systolic and diastolic function, and is able to potentially identify the different patterns of structural remodeling characterizing patients with rVT of different etiology.

## Introduction

Cardiac computed tomography (cCT) has impressively evolved in the last years, following the continuous improvement in temporal and spatial resolution. Nowadays, cCT is commonly used in the assessment of coronary artery disease [1]. Further emerging application comes from the possibility to characterize myocardial scars using late iodine enhancement (LIE) [2–4], based on the delayed wash-in and wash-out kinetic of the contrast media related to the presence of fibrosis and scar [5]. The detection of myocardial dense scars is fundamental to therapeutic decision-making, risk stratification, and therapeutic monitoring [3–7]; however, the assessment of diffuse remodeling of extracellular matrix is equally important. An increasing number of evidence indicate that diffuse myocardial fibrosis is a causative factor of myocardial dysfunction [8–11] and ventricular arrhythmias [12, 13].

Multiple non-invasive diagnostic strategies have been tested for the non-invasive assessment of diffuse extracellular matrix remodeling. T1 mapping assessed by cardiac magnetic resonance represents the new non-invasive standard of reference. The areas of diffuse interstitial fibrosis are characterized by higher native T1 when compared to normal myocardium [14]. Moreover, T1 mapping acquired before and after the injection of gadolinium allows measuring the relative expansion of the extracellular matrix [15], using the so-called parameter extracellular volume fraction (ECV). ECV can be calculated also on cCT with optimal correlation with cardiac magnetic resonance [16] and with the advantage of whole heart assessment [17]. ECV provides an overall assessment of the extracellular space expansion on average, without assessment of spatial heterogeneity of extracellular matrix. A heterogeneous remodeling of extracellular matrix was found to have a strong impact on the myocardial electrical instability and, thus, on the ventricular arrhythmic vulnerability [18, 19]. Extracellular matrix remodeling also plays an essential role in the determination of the mechanical properties of the myocardium. Changes in the extracellular matrix amount, composition, metabolism, and crosslinking are deeply involved in the increase of myocardial stiffness and in geometric remodeling of the left ventricle [20].

Although ECV has a strong prognostic meaning [21], its diagnostic value is limited. In fact, an increase of the ECV is not specific for a given diagnosis, because ECV expansion occurs in several cardiomyopathies.

The premise of this study is that the pattern and, particularly, the heterogeneity of the myocardial extracellular matrix remodeling could be extracted by texture analysis of LIE cCT images and that texture analysis of LIE may complement ECV, bacause it may reveal a non-invasive signature of different cardiac diseases.

Texture analysis is a mathematical computation that allows the extraction of complex quantitative features from images, increasing the potential informative value of radiological examinations. Texture features extracted from biomedical images are extensively explored for “radiomic-phenotypization” of different solid tumors [22]. Moreover, some studies have suggested that a texture analysis may extract quantitative information about liver fibrosis from conventional CT images [23], as well as interstitial myocardial fibrosis from late gadolinium enhancement (LGE) magnetic resonance images [24].

The aim of this study was to extract the myocardial ECV and LIE first-order texture features from cCT of patients with recurrent ventricular tachycardia (rVT) and to compare the extracted radiomic features with left ventricle remodeling, systolic-diastolic function by echocardiography, and underlying cardiac disease.

## Materials and Methods

This is a single-center observational prospective study performed on 52 consecutive patients suffering from rVT, who underwent cCT for a combined assessment of coronary vessels and myocardial scars, in order to plan trans-catheter radio-frequency ablation. Three out of 52 patients were excluded due to previous cardiac surgery with myocardial patch implantation. One patient was excluded from the study because of low quality of the cCT images due to respiratory artifacts.

Transthoracic echocardiography was performed in all patients before cCT examination.

This study was approved by the Institutional Review Board of the San Raffaele Scientific Institute and all patients signed an informed consent form.

### cCT Protocol

cCT examinations were performed on a multi-detector CT (Brilliance 64; Philips Medical System; The Netherlands) scanner. Patients were prepared with intravenous injection of beta-blockers (atenolol 1–15 mg) in the case of HR > 65 bpm. Three patients underwent cCT during continuous intravenous infusion of lidocaine hydrochloride, in order to control ventricular arrhythmias.

cCT protocol included a pre-contrast (80 kVp) prospectively gated scan which was used to obtain baseline blood and myocardial attenuation, followed by two sequential contrast-enhanced acquisitions: an angiographic scan and a LIE scan. Angiographic scan was acquired during triphasic injection of high-iodine (iopromide 370 mg iodine/ml or iomeprol 400 mg iodine/ml) contrast agent (CA) (90 ml CA, 40 ml of mixed solution 30 % CA - 70 % saline, 40 ml saline). This injection was immediately followed by a second intravenous injection to reach the total dose of 0.6 mg iodine/kg of body weight [25]. LIE scan was acquired after 10 min with low voltage (80 kVp) in order to increase relative density of myocardial scars [26].

The angiographic and LIE scans were acquired with prospective gating (*n* = 27) in patients with HR ≤ 65 bpm and with retrospective gating with ECG-based tube current modulation (*n* = 21) in patients with HR > 65 bpm despite beta-blockers infusion. Regardless, the ECG-gating mode chosen, the detector collimation was 64 × 0.625 mm, the rotation time 0.42 s and images were reconstructed with a standard cardiac kernel (CB), with a thickness of 0.75 mm and 512 × 512 matrix.

According to the method proposed by the European Working Group for Guidelines on Quality Criteria in CT [27], the effective radiation dose of LIE scan was respectively 1.5 ± 1.1 mSv in the case of prospective gating and 3.6 ± 1.3 mSv in the case of retrospective gating.

### 3D cCT Segmentation

Myocardial left ventricle (LV) wall and cardiac chambers were semi-automatically segmented on the angiographic scan at 75 % of the cardiac cycle using a commercial software (IntelliSpace Portal v7.0, Philips Healthcare, The Netherlands), as previously described [28]. Left atrium volume (LAV) was calculated by 3D segmentation and voxel summation, excluding pulmonary vein. LV end-diastolic volume (EDV) was calculated using the 75 % phase LV volume and 75 % phase LAV, according to the prediction model developed by Khatri PJ, et al. [29]: EDV = (1.021 × 75 %-phase LV volume) + (0.259 × 75 %-phase LAV).

Dense scars were identified and segmented as previously described [28]. Briefly, they were visually recognized as areas of wall thinning (wall thickness < 5 mm on angiographic images) or as areas of late iodine enhancement [28]. All scans were realigned using angiographic scan as the reference volume, where mutual information has been used as the similarity measure in the registration process [28].

### Texture Features and ECV Extraction from the LV Remote Myocardium

Segmented scars were extracted from the LV wall volume and used for ablation planning, as previously described [28]. The remote myocardial wall, which remained after the dense scar subtraction, was analyzed using MATLAB (R2014a) for the extraction of the ECV and the first-order texture features.

All pixels greater than 250 and 470 HU were excluded from the analysis for baseline and LIE, respectively, as well as all pixels lower than 0 HU. The lower cut-off of 0 HU was empirically chosen in order to automatically exclude pixels corresponding to air or adipose tissue [30] coming from fine errors of segmentation potentially occurring along the epicardial border and to exclude dark streak artifacts from implantable cardioverter defibrillator (ICD) electro-catheters [28]. The higher cut-offs (> 250 HU in the precontrast scan and > 470 HU in the LIE scan) were empirically chosen to be higher than the maximum densities of soft tissues (+ 20 % over the maximum soft tissues densities), as previously implemented [28], in order to exclude bright streak artifacts from ICD. The exclusion of all the pixel outside the lower and higher cut-offs chosen allows exclusion of artifacts or non-myocardial pixel (air, fat) in the subsequent steps of image analysis and, hence, to eliminate potential errors in the extraction of ECV and texture parameters.

First-order texture features were calculated from the segmented remote myocardium on the LIE scan, after filtration with a Gaussian kernel of standard deviation 1 voxel to reduce the image noise [31] according to the method previously described [32]. Then, we extracted the parameters describing HU spatial frequency distribution in terms of general attenuation (energy), statistical randomness (entropy), central tendency (HU mean value, HU median value), and scattering/variability: standard deviation (SD) and mean absolute deviation (MAD).

Myocardial and blood pool HU were extracted from the baseline and LIE scan. ECV was calculated as follows:

$$ \frac{\Delta {HU}_{myo}}{\Delta {HU}_{blood}}\times \left(1- Hct\right) $$

where Hct is the hematocrit (measured the same day of cCT scan), ΔHU_myo_ is the change in myocardial HU attenuation after iodine injection, and ΔHU_blood_ is the change of blood HU mean attenuation after iodine injection.

Image post-processing was around 30 min per patient.

### Transthoracic Echocardiography

Ejection fraction (EF), end-diastolic diameter (EDD), and diastolic function of the LV were assessed. Diastolic function was determined through the evaluation of the mitral inflow pattern and scored in a 4-point scale (0: normal pattern, 1: impaired myocardial relaxation, 2: pseudo-normalized pattern, 3: restrictive pattern), according to the current recommendations [33].

### Statistical Analysis

Spearman’s rho was used for evaluating correlations between LIE texture features (HU mean, HU median, SD, MAD, energy, and entropy), ECV and clinical features (age, BMI, HR, presence of ICD), scan protocol (ECG-gating mode of the CT scan: prospective *vs* retrospective) functional and volumetric parameters by cCT (EDV, LAV), and echocardiography (EF, EDD, LV diastolic function).

Differences among groups were determined using ANOVA test, and *p* values were adjusted for multiple parameters.

The texture parameters which resulted significantly different in relation to the underlying cardiac disease were then categorized in classes defined by each tertile of their distribution in order to provide possible cut-offs. The association between the subgroups and the types of cardiac disease were assessed through Fisher’s test. Bonferroni’s correction was applied to account for multiple comparisons. A multivariate ordinal regression analysis was employed in order to evaluate whether the subgroups were predictive of heart diseases, when accounting for EDV, EF, and diastolic function. The final models were obtained using a backward selection procedure. The *p* values less than 0.05 were considered to be significant. All statistical analyses were performed using R 3.2.0 (http://www.R-project.org/).

## Results

### Clinical and LV Volumetric and Functional Parameters

A total of 48 consecutive patients with rVT (91 % male; 9 % female) were evaluated. According to previous medical records, echocardiography, invasive coronary angiography, and in most of cases, cardiac magnetic resonance, patients were classified as follows: 23 out of 48 patients (48 %) suffered from post-ischemic dilated cardiomyopathy (ICM), 9 out of the 48 patients (19 %) had idiopathic dilated cardiomyopathy (IDCM), 11 (23 %) had myocardial scars from a previously healed myocarditis (MYO), and 5 (10 %) suffered from idiopathic ventricular tachycardia (IVT) without any evidence of structural heart disease. The baseline characteristics of enrolled patients are listed in Table 1. MYO patients were slightly younger than ICM and IDCM patients. As expected, hyperlipidemia was more common in ICM than MYO and IVT patients. Incidence of hypertension and diabetes was not significantly different among groups, as well as the incidence of ICD. Not surprisingly, ICM and IDCM patients had more dilated and dysfunctional LV with significantly higher EDV and reduced EF in comparison with both MYO and IVT. Moreover, ICM and IDCM patients had worse diastolic function and larger LAV in comparison with MYO and IVT (Table 1).

**Table 1:** Baseline characteristics

	All patients	ICM	IDCM	MYO	IVT	*p* value
Age, year	61 ± 15	67 ± 8*	67 ± 6°	46 ± 18*^,^°	56 ± 20	0.00008* 0.02°
Sex, female/male	4/44	0/23	2/8	2/11	0/5	n.s.
BMI, kg/m^2^	26 ± 3	26 ± 3	27 ± 4	24 ± 2	25 ± 3	n.s.
Hypertension	23/48	9/23	2/9	3/11	3/5	n.s.
Diabetes	7/48	5/23	2/9	0/11	0/5	n.s.
Hyperlipidemia	18/48	9/23*°	2/9	1/11°	0/5*	0.03* 0.01°
ICD (pts)	36/48	20/23	8/9	6/11	2/5	n.s.
Heart rate (bpm)	66 ± 10	64 ± 9	74 ± 14	63 ± 10	63 ± 10	n.s.
EF, %	43 ± 13	38 ± 10 *^,§^	34 ± 11°^,¶^	52 ± 7*^,^°	58 ± 13 ^§,¶^	0.003*; 0.002°; 0.001^§^; 0.0005^¶^
EDD, mm	59 ± 9	61 ± 8^§^	66 ± 7°^,¶^	54 ± 7°	47 ± 8^§,¶^	0.004°; 0.002^§^; 0.0001^¶^
EDV, ml	276 ± 88	301 ± 87*^,§^	332 ± 35*^,§^	218 ± 51*^,^°	184 ± 35^§,¶^	0.025*; 0.001°; 0.046^§^; 0.003^¶^
Diastolic function Normal Impaired Pseudonormalized Restrictive	15 24 7 2	* 1 16 6 0	° 2 4 1 2	*^,^° 9 2 0 0	3 2 0 0	0.0005*; 0.002°
Left atrium volume, ml	133 ± 62	133 ± 39*	193 ± 98*^,^°^,¶^	102 ± 32°	91 ± 42^¶^	0.04*; 0.003°; 0.02^¶^

Values are mean ± SD, unless otherwise indicated. Statistically significant diferences are indicated by symbols near to each variable; the relative *p* value is reported in the last column and labeled with the same symbol

*ICM* ischemic cardiomyopathy, *IDCM* idiopathic dilated cardiomyopathy, *MYO* post-myocarditis syndrome, *IVT* idiopathic ventricular tachycardia, *BMI* Body Mass Index, *ICD* implantable cardioventer defibrillator, *EF* left ventricular ejection fraction, *EDD* left ventricular end diastolic diameter, *EDV* left ventricular end diastolic volume, *LAV* left atrium volume, *n.s.* no statistically significant difference

### Texture Features and ECV Correlations with Functional and Volumetric Parameters from cCT and Echocardiography

Values of texture features, ECV, and relative differences among groups are reported in Table 2.

**Table 2:** Texture features of late iodine enhanced (LIE) images and volumetric extracellular volume fraction (ECV) from cardiac CT examination

	All patients	ICM	IDCM	MYO	IVT	*p* value
ECV	42 ± 7	44 ± 6	44 ± 4	38 ± 6	39 ± 9	n.s.
Energy (× *e*^10^)	8 ± 6	8 ± 6	10 ± 6	8 ± 6	7 ± 6	n.s.
Entropy	6 ± 0.3	6 ± 0.3	6 ± 0.4	6 ± 0.4	6 ± 0.4	n.s.
HU mean	200 ± 48	205 ± 42	227 ± 9	183 ± 60	168 ± 68	n.s.
HU median	206 ± 51	211 ± 44	236 ± 9	186 ± 60	169 ± 71	n.s.
SD	42 ± 12	45 ± 8*,**	49 ± 14°,°°	34 ± 9*,°	29 ± 6**,°°	0.01*; 0.004°; 0.008**; 0.003°°
MAD	29 ± 8	31 ± 6*,**	34 ± 11°,°°	23 ± 6 *,°	20 ± 3**,°°	0.02*; 0.007°; 0.02**; 0.005°°

Data are mean ± SD unless otherwise indicated. Statistically significant diferences are indicated by symbols near to each variable; the relative *p* value is reported in the last column and labeled with the same symbol

*n.s.* no statistically significant difference

ECV correlated with texture parameter of general attenuation (ECV *vs* energy: rho = 0.5650, *p* < 0.0001) and parameters of central tendency (ECV *vs* HU mean value: rho = 0.5741, *p* < 0.0001, ECV *vs* HU median: rho = 0.5068, *p* = 0.0002). No significant correlations were found between ECV and texture features of randomness and variability.

Correlations between ECV and texture features with clinical parameters, cCT scan protocol, LV volumetric and functional data, and LAV are reported in Table 3. Briefly, age correlated only with ECV; BMI, heart rate, presence/absence of ICD, and ECG-gating mode of the CT scan did not correlate either with ECV or texture parameters. ECV and some texture parameters (HU mean, HU median, SD, and MAD) correlated directly with EDV and inversely with EF. The same texture features (HU mean, HU median, SD, and MAD) correlated with EDD. ECV and texture parameters of scattering (SD and MAD) correlated with LAV. Only texture parameters of scattering (SD and MAD) correlated with LV diastolic function.

**Table 3:** Correlation between ECV, LIE texture features, clinical, functional, and volumetric parameters from cCT and echocardiography

	ECV	Energy	Entropy	HU mean	HU median	SD	MAD
Age, year	rho = 0.3748 *p* = 0.0087	n.s.	n.s.	n.s.	n.s.	n.s.	n.s.
BMI	n.s.	n.s.	n.s.	n.s.	n.s.	n.s.	n.s.
ICD	n.s.	n.s.	n.s.	n.s.	n.s.	n.s.	n.s.
Heart rate	n.s.	n.s.	n.s.	n.s.	n.s.	n.s.	n.s.
ECG-gating mode	n.s.	n.s.	n.s.	n.s.	n.s.	n.s.	n.s.
EF, %	rho = − 0.3335 *p* = 0.0205	n.s.	n.s.	rho = − 0.4112 *p* = 0.0037	rho = − 0.4703 *p* = 0.0007	rho = − 0.3943 *p* = 0.0056	rho = − 0.3889 *p* = 0.0063
EDD, mm	n.s.	n.s.	n.s.	rho = 0.3490 *p* = 0.0151	rho = 0.4190 *p* = 0.0030	rho = 0.3830 *p* = 0.0072	rho = 0.3489 *p* = 0.0151
EDV, ml	rho = 0.3776 *p* = 0.0081	n.s.	n.s.	rho = 0.3183 *p* = 0.0279	rho = 0.3678 *p* = 0.0105	rho = 0.3490 *p* = 0.0105	rho = 0.3108 *p* = 0.0320
Diastolic function	n.s.	n.s.	n.s.	n.s.	n.s.	rho = 0.3837 *p* = 0.0071	rho = 0.3330 *p* = 0.0208
LAV, ml	rho = 0.3120 *p* = 0.0309	n.s.	n.s.	n.s.	n.s.	rho = 0.4344 *p* = 0.002	rho = 0.3575 *p* = 0.0126

Diastolic function is scored in a 4-point scale (0: normal pattern, 1: impaired myocardial relaxation, 2: pseudo-normalized pattern, 3: restrictive pattern)

*BMI* Body Mass Index, *ICD* implantable cardioventer defibrillator, *EF* ejection fraction, *EDD* left ventricular end diastolic diameter, *EDV* left ventricular end diastolic volume, *LAV* left atrium volume, *n.s.* no statistically significant correlation

### Association Between Radiomic Features of Remote Myocardium Heterogeneity and Heart Disease Underlying rVT

**Fig. 1:**
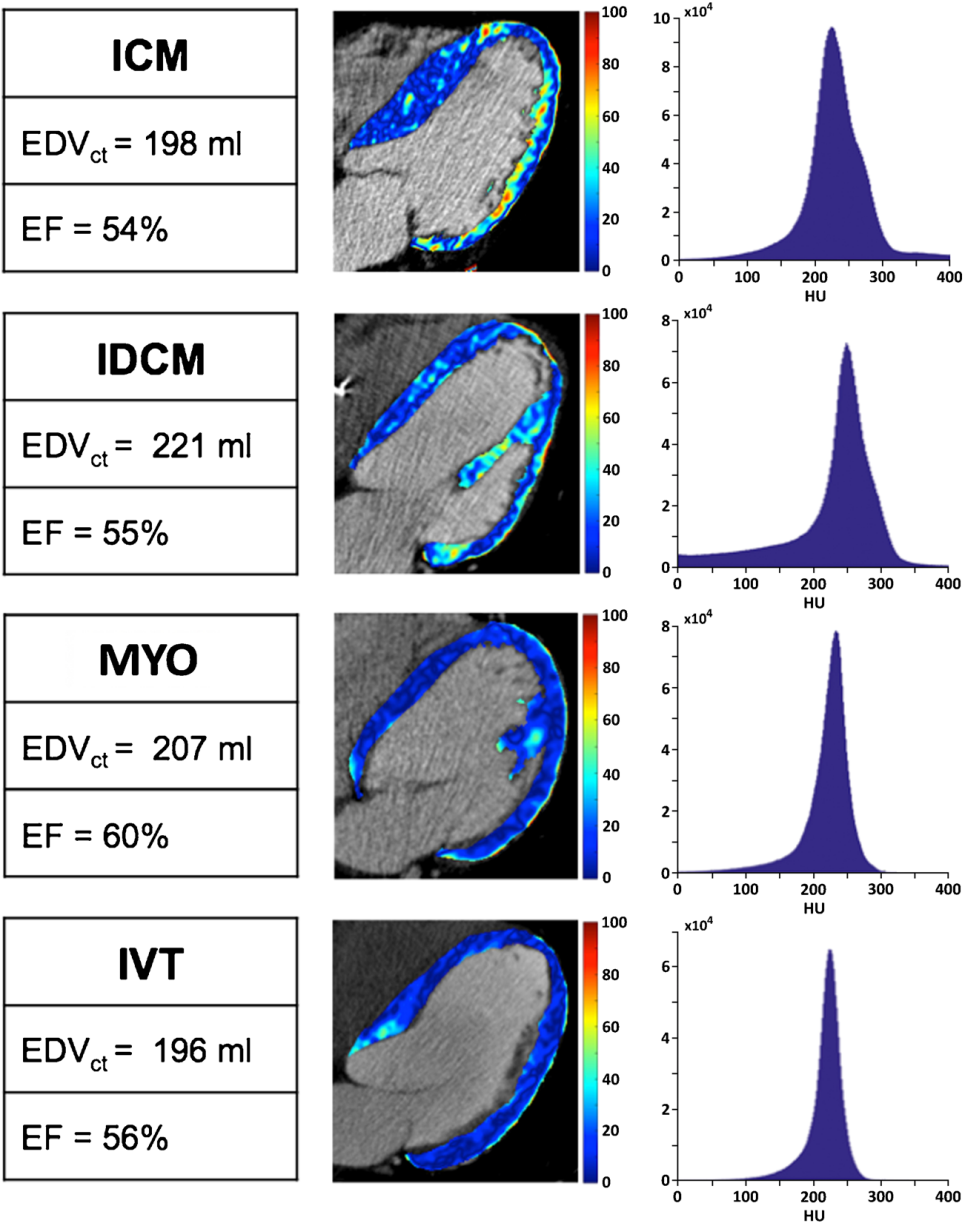
Color-coded HU magnitude maps of Late Iodine Enhancement (LIE) images, overlapped with the angiographic scan, are reported for one patient of each group. Reported maps allow to visually assess the higher heterogeneity of the non-scarred myocardium in ischemic cardiomyopathy (ICM) and idiopathic dilated cardiomyopathy (IDCM) patients, rather than in those with post-myocarditis syndrome (MYO) and idiopathic ventricular tachycardia (IVT). The respective histograms showed a wider dispersion of HU value, suggesting higher heterogeneity in both ICM and IDCM patients rather than in the patients with MYO and IVT, regardless of the similar volumes and function of left ventricle (LV).

Magnitude maps of patients with similar LV volume and function, but different underlying cardiac disease, showed different degree of myocardial heterogeneity (Fig. 1).

**Fig. 2:**
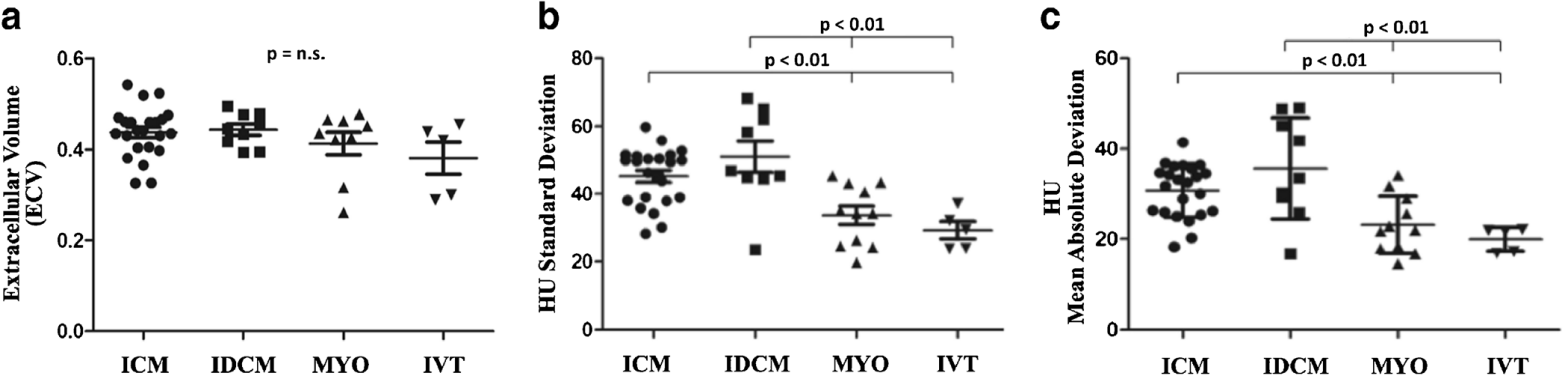
Single-patient scatter plots of **a** extra-cellular volume fraction (ECV), **b** standard deviation (SD), and **c** mean absolute deviation (MAD) texture features. ECV was not significantly different among groups. SD and MAD were significantly higher in ischemic cardiomyopathy (ICM) and idiopathic dilated cardiomyopathy (IDCM) patients compared with both patients with post-myocarditis syndrome (MYO) and idiopathic ventricular tachycardia (IVT).

As showed in Fig. 2, SD and MAD resulted significantly higher in ICM and IDCM patients compared with MYO and IVT (Table 2), despite no significant difference in the ECV (Table 2).

**Fig. 3:**
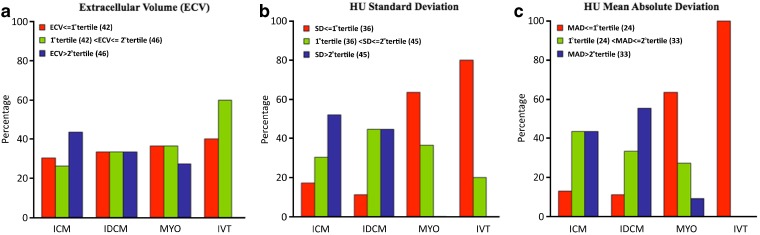
Bar plots of prevalence of each type of heart disease according to **a** ECV, **b** SD, and **c** MAD values categorized in tertile of their distribution. Numerical values indicating the first and second tertiles of each parameter are reported in round brackets. Distribution of heart disease was significantly different for SD (*p* = 0.0168) and MAD (*p* = 0.0070), while it was not significantly different for ECV (*p* = 1.000). ECV values were comparable among classes. SD and MAD showed significantly lower values (mainly G first tertile of their distribution) in MYO and IVT patients. *p* value was adjusted according to the Bonferroni correction. ICM ischemic cardiomyopathy, IDCM idiopathic dilated cardiomyopathy, MYO post-myocarditis syndrome, IVT idiopathic ventricular tachycardia.

The percentage of patients in the first, second, and third tertiles of SD and MAD showed significant differences (*p* value 0.0168 and 0.0070, respectively) in relation to the underlying cardiac disease (Fig. 3). For instance, 100 % of MYO and IVT patients were in the first and second tertile for SD. Conversely, most of ICM and IDCM patients (83 and 89 %, respectively) were in the second and third tertile (Fig. 3).

In multivariate ordinal regression analysis, only SD and MAD were significantly different among cardiac disease, independently by the degree of volumetric remodeling of the LV (EDV, EDD), systolic (EF), and diastolic function.

ECV, energy, entropy, HU mean, and HU median were not significantly associated with different cardiac diseases (*p* = 1.000, *p* = 1.000, *p* = 1.000, *p* = 1.000, *p* = 0.2625, respectively).

## Discussion

Nowadays, cCT is a diagnostic tool widely used to study patients with suspected coronary artery disease. The iodinated contrast agent, usually injected to assess the epicardial vessels, progressively diffuses in the extracellular space, reaching in few minutes a condition of balance between blood and interstitial compartment. In this equilibrium phase, the concentration of the contrast agent within a given tissue is a function of the relative proportion between the extracellular and intracellular space. Hence, a cCT study may provide valuable information about the extracellular volume fraction, when the angiographic scan is followed by a LIE scan. Nacif et al. demonstrated the possibility to extract ECV from low-dose CT, obtaining a good correlation with magnetic resonance [16], with the further advantage of a whole-heart ECV quantification [17].

The hypothesis that inspired our study is that LIE images, usually acquired to detect myocardial scar, may include information about the pattern of remote myocardium extracellular matrix remodeling. We believe that this information could be extracted by texture analysis of LIE images that, to the best of our knowledge, was never performed before.

We extracted six texture parameters and ECV from the remote myocardium of 48 patients suffering from the rVT of variable etiology. Among texture parameters, we measured descriptors of the general attenuation (energy) and statistical randomness (entropy), parameters of central tendency (HU mean and HU median), and parameters of scattering/variability (standard deviation and mean absolute deviation). In agreement with the previous study by Nacif [17], ECV correlated directly with EDV and inversely with the EF and was not significantly associated with diastolic function. Texture parameters of general attenuation and central tendency correlated with ECV, and as expected, they correlated directly with EDV and inversely with EF.

Texture parameters of general attenuation and central tendency provide information about the global accumulation of iodine in the extracellular matrix. The higher the HU mean and median are, the higher the accumulation of iodine in the extracellular matrix is. Hence, HU mean and median result correlated to ECV because they give similar information, and similarly to ECV, HU mean and median were not significantly different across patient groups, demonstrating a similar increase of the extracellular myocardial space in ICM, IDCM, MYO, and IVT.

Only parameters of HU variability (SD and MAD) were correlated with diastolic function score, demonstrating that when the HU variability is higher, the diastolic function is worsen. Moreover, we found that SD and MAD values were significantly different in relation to the underlying cardiac disease. In fact, patients suffering from IVT and MYO had SD values under the 66th percentile in 100 % of the cases, and most of them were under the 33rd percentile (80 and 64 % respectively), whereas only 17 and 11 % of ICM and IDCM patients had SD values under the 33rd percentile (Fig. 3). This was similar to the distribution of MAD values, with 100 % of IVT and MYO cases under the 66th percentile, while only 13 and 11 % of ICM and IDCM patients were under the 33rd percentile (Fig. 3). These associations were not driven by the differences in LV dilatation or function as demonstrated by the multivariate ordinal regression analysis.

Although we have found higher SD and MAD in the patients with worsen diastolic function as well as in patients affected by cardiac diseases typically associated with loss of LV elasticity and compliance (ICM and IDCM), our results cannot demonstrate a direct cause-effect relationship between texture parameters of variability and diastolic function impairment. Nevertheless, the significant correlation of diastolic function with parameters of variability but not with ECV suggests that the structural architecture of ECM remodeling could be more important than the extracellular space enlargement *per se* in affecting the wall stiffness, even if this hypothesis needs to be confirmed by future investigations.

Considering the emerging role of SD as texture parameter for non-invasive characterization of tissue heterogeneity in cancer [34], our results suggest that texture analysis of LIE images may provide information about the heterogeneity of remote myocardium structural remodeling that seems able to characterize different cardiac diseases when the ECV fails. Different degrees of myocardium heterogeneity revealed by LIE texture analysis might reflect known histopathological features characterizing different cardiomyopathies. Previous histopathological studies demonstrated that a pathognomonic feature of the remodeling of remote myocardium in ICM was represented by the presence of patchy (few millimeters) foci of fibrous tissue, associated with diffuse, perivascular, and interstitial fibrosis [8, 35]. These fibrotic alterations undetected by LGE acquisition account for more than two-thirds of the fibrous tissue found in the ICM, whereas the infarct scar constitutes only one third [8] of it. Similarly, in IDCM, myocardial fibrosis occurs mainly in the form of small areas of interstitial and replacement fibrosis that may also be associated with perivascular pattern [36]. On the other hand, patients healed from acute myocarditis with a normal or close to normal ventricular volumes and function as the patients included in the MYO group of our study were expected to have less heterogeneity of the extracellular matrix outside the macroscopic scars resulting from the acute phase of disease, as well as the patients without overt structural substrate of rVT (IVT patients).

In addition, recent literature provides a proliferation of evidence indicating that the diffuse structural remodeling is a key factor in the genesis of vulnerability to electrical re-entry circuits [37, 38] and the degree of heterogeneity of diffuse fibrosis is potentially associated with risk of arrhythmia onset [18]. Hence, the non-invasive assessment of the texture of diffuse fibrosis using cCT images could be proposed for future studies investigating its potential role in arrhythmogenic risk stratification.

In conclusion, our results suggest that texture analysis of LIE images may provide distinctive information about microstructural myocardial changes, which may help in the discrimination of cardiac diseases, simply by a computational analysis of conventional images acquired in the clinical routine.

However, this suggestion needs to be reassured and supported by future experimental studies which will emphasize on tackling several limitations of our study, such as the relative small sample size, the focus only on patients with ventricular tachycardia, the lack of reproducibility tests, although texture analysis of CT images has been extensively validated and reproduced in many studies [39], and the absence of comparison with magnetic resonance or histology. Unfortunately, most of the subjects in our study had ICD, and it is known that T1 mapping accuracy may be significantly affected by the presence of cardiac devices [40]. Furthermore, histopathological validation, although needed, may give uncomplete and potentially deceptive results if based on myocardial bioptic samples from patients, due to sampling error bias. Therofore, we rely on future studies upon animal models for a detailed and accurate definition of exact structural or biological meaning of myocardial texture features.

## Conclusions

Our results suggest that texture parameters describing the scattering of densities within the myocardium may provide a deeper characterization of the micro-architectural changes occurring in the extracellular myocardial space, impacting on the biomechanical properties of the left ventricle and potentially useful in the characterization of myocardial diseases.
